# Dementia-related volumetric assessments in neuroradiology reports: a natural language processing-based study

**DOI:** 10.1136/bmjopen-2024-092459

**Published:** 2025-09-28

**Authors:** Adam John Mayers, Angus Roberts, Ashwin V Venkataraman, Christopher Booth, Robert Stewart

**Affiliations:** 1 King’s College London, Institute of Psychiatry Psychology & Neuroscience, London, UK; 2 Guy's and St Thomas’ NHS Foundation Trust, London, UK; 3 South London and Maudsley NHS Foundation Trust, London, UK

**Keywords:** Feasibility Studies, Natural Language Processing, Magnetic resonance imaging, RADIOLOGY & IMAGING, Dementia

## Abstract

**ABSTRACT:**

**Objectives:**

Structural MRI of the brain is routinely performed on patients referred to memory clinics; however, resulting radiology reports, including volumetric assessments, are conventionally stored as unstructured free text. We sought to use natural language processing (NLP) to extract text relating to intracranial volumetric assessment from brain MRI text reports to enhance routine data availability for research purposes.

**Setting:**

Electronic records from a large mental healthcare provider serving a geographic catchment of 1.3 million residents in four boroughs of south London, UK.

**Design:**

A corpus of 4007 de-identified brain MRI reports from patients referred to memory assessment services. An NLP algorithm was developed, using a span categorisation approach, to extract six binary (presence/absence) categories from the text reports: (i) global volume loss, (ii) hippocampal/medial temporal lobe volume loss and (iii) other lobar/regional volume loss. Distributions of these categories were evaluated.

**Results:**

The overall F1 score for the six categories was 0.89 (precision 0.92, recall 0.86), with the following precision/recall for each category: presence of global volume loss 0.95/0.95, absence of global volume loss 0.94/0.77, presence of regional volume loss 0.80/0.58, absence of regional volume loss 0.91/0.93, presence of hippocampal volume loss 0.90/0.88, and absence of hippocampal volume loss 0.94/0.92.

**Conclusions:**

These results support the feasibility and accuracy of using NLP techniques to extract volumetric assessments from radiology reports, and the potential for automated generation of novel meta-data from dementia assessments in electronic health records.

**Strengths and limitations of this study**

- To our knowledge, there has been no prior published research on the automated extraction of terms relating specifically to volumetric assessments from brain MRI reports.
- This study uses natural language processing to extract mentions of dementia-related volumetric assessments from brain MRI reports which would not be feasible in a large dataset.
- Reports are from patients referred for imaging from a single healthcare provider and the reports will have been written by a comparatively small number of neuroradiologists although over a long time period (13 years).
- The majority of the reports are addressing the same clinical question and the model would not be generalisable to other radiology reports.
- The model does not seek to capture longitudinal or comparative data. Some of the reports only mentioned that there was no progressive atrophy, implying a consideration of previous reports, and there was no means to evaluate what this meant for the scan in question.

## Introduction

### Background

 Brain MRI scans are performed on many patients being evaluated for a dementia diagnosis. The radiology report forms an important part of the patient health record, with the radiologist conventionally composing a free text document describing their findings following their interpretation of the images. In the context of dementia, this report is likely to include mentions of the presence or absence of generalised, regional or more specific focal areas of volume loss, as these findings in conjunction with the clinical picture help to guide diagnosis. For example, the presence of hippocampal atrophy is an important finding in the context of suspected Alzheimer’s type dementia,^1^ as is the regional vulnerability to neurodegeneration across a variety of different dementia-related processes.^2^ Electronic health records (EHRs) provide important opportunities for research using large historic cohorts, although research questions are limited by the availability of data in a structured format for analysis. Extracting mentions of intracranial volumes from the radiology report would allow structured data to be generated and used for cohort assembly and analysis for downstream research.

### Natural language processing in radiology

Natural language processing (NLP) harnesses computational techniques to scrutinise both written and spoken language. Supervised approaches include rule based and/or machine learning based strategies, where the latter involve the deployment of methods such as deep learning and necessitate large volumes of data for training, validation and testing. The implementation of EHRs has made large volumes of digital data readily available for the application of NLP techniques, and these are increasingly being used within medicine with a wide variety of applications.^3^ This includes implementation within the field of radiology, with multiple systematic reviews demonstrating increasing usage of NLP techniques.^4^,^6^ Applications include diagnostic surveillance, cohort building for research studies, query-based case retrieval, quality assurance, clinical support^7^ and automated protocolling.^8^

### Natural language processing in neuroimaging and dementia

Even within neuroradiology, there are a large number of uses for NLP, such as extraction from reports of any mentions of ischaemic stroke and their acuity/location^9^, detection of white matter hyperintensities and silent brain infarcts,^10^ or more broad detection of stroke, haemorrhage, tumour and atrophy.^11^ In the dementia field, machine learning techniques have been used to model the progression of Alzheimer’s disease,^12 13^ and more specifically, NLP techniques have been applied to several areas including diagnosis from speech^14^ or extracting symptomology from the clinical record.^15^

To our knowledge, there are currently no papers focussing on the automated extraction of terms relating to volumetric assessments, as would be of particular use in a dementia context. We therefore sought to develop an automated method for labelling and extracting dementia-related volumetric assessments from neuroimaging reports in a large EHR database using machine learning techniques. We evaluated the performance of our method on a dataset of MRI reports from patients referred for imaging from memory clinic and compared it with manual review of original text.

## Methods

### Corpus selection and preprocessing: the South London and Maudsley mental health case register

The South London and Maudsley NHS Foundation Trust (SLaM) provides mental health and dementia care services to around 1.3 million residents in its geographic catchment of four south London boroughs (Lambeth, Southwark, Lewisham and Croydon). SLaM adopted EHRs across all of its services in 2006. In 2007–2008, the Clinical Record Interactive Search (CRIS) application was developed with funding from the British National Institute for Health Research.^16^ CRIS generates a research database containing pseudonymised versions of de-identified patient records on more than 250 000 patients and 3.5 million documents. Since its development, the data have been substantially enhanced through external linkages and NLP.^17^ CRIS and associated data resources have received ethical approval for secondary analyses (Oxford Research Ethics Committee C, reference 23/SC/0257).

**Figure 1:**
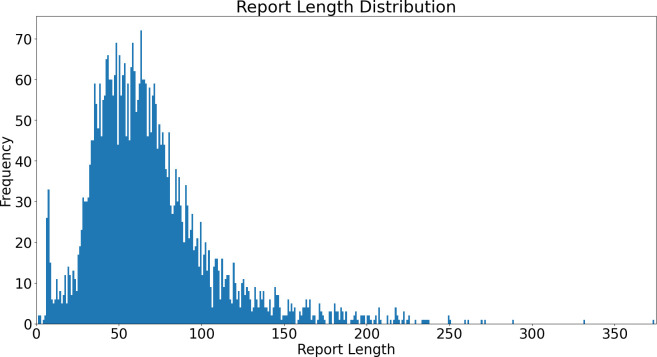
Distribution of length of report in words.

A total of 5672 radiology events were extracted from the radiology information system data for patients referred from memory assessment services between 5 August 2008 and 25 October 2021. After the exclusion of events where there was no associated report (ie, where the event was cancelled, the patient did not attend or there was no image acquisition for another reason), there were 4007 free text records. The total number of words in the corpus was 280 621 over 19 304 sentences, with a mean of 14.5 words per sentence. The mean number of words per report was 68 (median=61), with a report length ranging from 1 to 373 words (figure 1).

### Technique

A span categorisation technique was used rather than a named entity recognition approach. Span categorisation^18^ involves labelling a collection of contiguous tokens (span) as belonging to category, where any span can have an arbitrary number of labels, including those that are nested or overlapping. This is in contrast to labelling individual tokens as belonging to a particularly named entity (as in Named Entity Recognition approaches, or NER). There are a number of advantages to a span categorisation approach for this specific task. First, the terms of interest in these types of reports are often overlapping (eg, ‘no lobar, regional, or hippocampal volume loss’) and a span categorisation approach allows multilabel classification at a token level meaning that overlapping or nested terms of interest can be represented easily. Second, the mentions of volumetric assessment are generally longer than would be considered as a single named entity (eg, ‘severe bilateral hippocampal and medial temporal lobe volume loss’) and as such do not have as clear token boundaries as would be required for a named entity recognition approach. Third, negation statements are also frequently distant from the term itself (including within a preceding sentence), which is more challenging for named entity recognition approaches. Finally, it allows broad inclusion within categories (eg, all other named areas of the brain that are not the hippocampus) that would be challenging to encompass with an NER approach.

We identified three key types of volume loss that were relevant to this use case—generalised atrophy, medial temporal lobe/hippocampal atrophy and other areas of regional atrophy. While the ‘regional’ category could be subdivided further (eg, ‘frontal volume loss’, ‘parietal volume loss’), the negation of volume loss was usually documented as ‘no regional or lobar atrophy’, and therefore, it was decided to model the simpler absence vs presence of regional volume loss. While this could have been modelled as absence of regional volume loss vs *n* categories of regional volume loss (where *n* is an arbitrary list of brain regions), the list of regions would be difficult to decide on without first reading the entire dataset, and increasing the number of categories increases the challenge of the annotation task and reduces the number of instances of each category in the dataset.

Therefore, six target categories were proposed:

- Global volume loss—present.
- Global volume loss—absent.
- Regional volume loss—present.
- Regional volume loss—absent.
- Hippocampal volume loss—present.
- Hippocampal volume loss—absent.

### Annotation and description of labelled spans

The guidelines for annotation were written following examination of approximately 500 randomly selected reports. Generally, the annotated span would include any laterality or descriptor of severity (eg, ‘severe bilateral hippocampal volume loss’) and was based only on what could be taken as objective from the report. In cases where a finding was related to a patient’s age (which was neither known to the model nor the annotator), this qualifying language was excluded and only the phrase which could be taken as objective was annotated. As an example, ‘global volume loss in keeping with the patient’s age’ would have ‘global volume loss’ categorised as such. However, ‘brain volume is normal for the patient’s age’ would have ‘brain volume is normal’ categorised as such. The annotation guidelines are supplied as online supplemental material.

The annotation was performed using the prodigy software library.^19^ Both annotators were senior clinical radiology registrars. Inter-annotator agreement was calculated using the F1 measure, that is, assuming one annotator was the ‘gold standard’ and measuring the other annotator’s performance against this. This approach was used as Cohen’s kappa (the standard inter-annotator agreement used for classification tasks) does not function well in this instance, as the vast majority of tokens in the dataset are not labelled at all, resulting in a very unbalanced dataset and artificially inflating the level of kappa agreement.^20^ Inter-annotator agreement using exact matches (ie, the presence of a span, its label, and both the start and end tokens) was 0.61 (95% CI 0.59 to 0.63). Using a relaxed definition of a match, as in Wang *et al*^21^ (ie, the presence of a span, its label and at least overlapping token boundaries), this rose to 0.83 (0.82–0.85).

**Figure 2:**
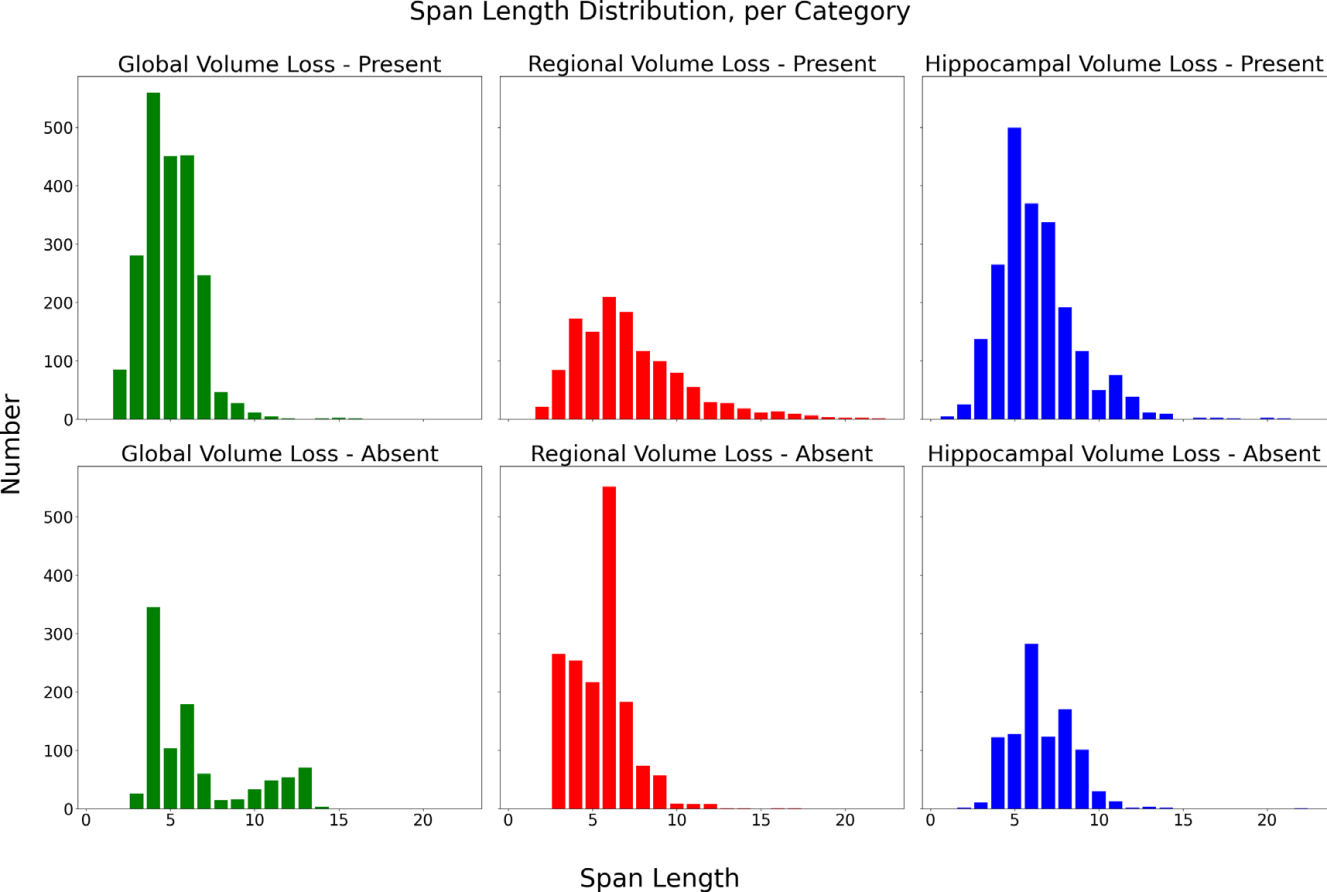
Distribution of lengths of labelled spans, per span category.

Following annotation of the full 4007 reports, 9154 total span examples were generated. Each category contained between 951 and 2165 examples, and span length ranged from 1 to 22 tokens with a median span length of 6. Distributions are visualised in figure 2.

Both span and boundary distinctiveness values were obtained using the spaCy library which calculates the Kullback–Leibler divergence of the span and boundary tokens in comparison to the remainder of the text.^22^ Average span distinctiveness and boundary distinctiveness were 1.84 and 1.42 respectively, with some variability between the different categories (table 1).

**Table 1:** Average span length, span distinctiveness and boundary distinctiveness per category

Category	Average span length	Span distinctiveness	Boundary distinctiveness
Global volume loss—present	4.71	1.91	1.33
Global volume loss—absent	5.89	1.87	1.76
Regional volume loss—present	6.48	1.38	1.20
Regional volume loss—absent	5.21	2.23	1.59
Hippocampal volume loss—present	5.88	1.65	1.27
Hippocampal volume loss—absent	6.37	2.04	1.63
Weighted average	**5.62**	**1.84**	**1.42**

Bold values are the weighted average of the rows above.

### Internal report inconsistency

As data are labelled at a word/token level, it is possible for a report to contain ‘contradictory’ labels. For some instances, this is appropriate: for example, both ‘left hippocampal atrophy’ and ‘right hippocampus is normal’ can appear within the same report. However, there can also be genuine inconsistencies within the report for example, both ‘no lobar or regional atrophy’ and ‘there is generalised widening of the parietal sulci’. However, this reflects a degree of inconsistency in the initial report rather than an error in the labelling. The overall number of reports containing conflicting labels, however, was small (141 reports, 3.5%) and this issue was not considered further in any analysis and these reports were not excluded.

### Model development

The 4007 total annotated reports were then subdivided, with 80% (3206 reports) being used for model development and 20% (801) being kept as a separate holdout test set. The spaCy Python library was used for model development of the algorithm.^23^ K-fold cross-validation with five folds was implemented^24^ to evaluate model performance for the purposes of base model selection and for further hyperparameter tuning.

The model was initially trained on each of spaCy’s publicly available English language pipelines (both convolutional neural networks and transformer architectures), including those trained on general web text as well as those from the scispacy library trained on a scientific corpus. The best performing model with default parameters was the transformer model trained on general web text (‘en_core_web_trf’^25^), and this was used as the basis for further model development. The pipeline consisted only of the tokenisation, transformer and span categorisation components (‘spancat’, which itself contains the suggester function for candidate spans, and a labeller that predicts zero or more labels for per candidate span). Including other pipeline components such as part of speech tagging, parsing and other available pipeline components did not improve performance and increased both resource usage and training time. After base architecture selection, hyperparameter tuning was performed (please see GitHub for spaCy configuration files^24^).

## Results

Against the holdout test set, the model’s F1 score averaged over all six categories was 0.89, with precision of 0.92 (95% CI 0.90 to 0.93) and recall of 0.86 (95% CI 0.84 to 0.87). Performance varied across the categories, with five of the categories in the 0.85–0.95 range, and regional volume loss being a relative outlier with F1 score of 0.68 (table 2).

**Table 2:** Precision, recall and F1 score for each span category

Category	Precision (95% CI)	Recall (95% CI)	F1 score
Global volume loss—present	0.95 (0.93 to 0.97)	0.95 (0.92 to 0.97)	0.95
Global volume loss—absent	0.94 (0.90 to 0.97)	0.77 (0.70 to 0.82)	0.85
Regional volume loss—present	0.80 (0.74 to 0.85)	0.58 (0.52 to 0.65)	0.68
Regional volume loss—absent	0.91 (0.88 to 0.94)	0.93 (0.90 to 0.95)	0.92
Hippocampal volume loss—present	0.90 (0.87 to 0.93)	0.88 (0.85 to 0.91)	0.89
Hippocampal volume loss—absent	0.94 (0.90 to 0.97)	0.92 (0.88 to 0.95)	0.93

### Inferred prevalence of specific types of atrophy

By using the presence of at least one instance of a span category to label the whole report, we can infer the reported prevalence of each type of atrophy in the cohort (table 3).

**Table 3:** Total number of reports containing at least one instance of a span category, and as a percentage of the whole cohort

Category	Total	Prevalence
Global volume loss—present	1852	46.2%
Global volume loss—absent	955	23.8%
Regional volume loss—present	952	23.8%
Regional volume loss—absent	1607	40.1%
Hippocampal volume loss—present	1488	37.1%
Hippocampal volume loss—absent	975	24.3%

## Discussion

We developed an application capable of extracting mentions of volumetric assessment from radiologist’s unstructured plain text brain MRI reports, to enable enrichment of routinely collected data for use in research. The algorithm was able to perform this task at good levels of accuracy against manually annotated source text, with an overall F score of 0.89, and a precision and recall of 0.92 and 0.86 respectively. The best performing category was the ‘global volume loss – present’ category (F1 score of 0.95), likely a function of both number of occurrences (this was the category with the most examples) and more stereotyped phrasing.

The algorithm relatively underperforms on the ‘regional volume loss - present’ category (F1 score of 0.68). This is somewhat expected given the underlying problem; if regional volume loss is present, then any anatomical term may be mentioned. This inherently makes this category much more heterogeneous in comparison to mentions of generalised volume loss or hippocampal volume loss, both of which need a much smaller vocabulary to describe. If regional volume loss is absent, then this similarly can only be described in a much more limited number of ways than its presence. This is in part reflected in the data, with the ‘regional volume loss – present’ category having the broadest range of span length (2–22) and the lowest span distinctiveness (1.38).

### Strengths and limitations

To our knowledge, there has been no published research on the automated extraction of terms relating specifically to volumetric assessments from brain MRI reports, as would be of particular use in a dementia context. We accurately extracted these terms using a model trained on a moderately sized dataset (more than 3200 radiologist-labelled reports out of the full annotated dataset of 4007). This model can now be used to extract these measures on a population-level basis and be used for cohort identification and comparison with other measures from age to performance on cognitive testing. Additionally, this model has demonstrated that this approach could be easily used to develop similar algorithms for different clinical problems with the appropriate labelled text.

Limitations include the use of reports from patients referred for imaging from a single healthcare provider; therefore, the reports will have been written by a comparatively small number of subspecialised neuroradiologists. This may have limited the variety of reports and writing styles that were available to train and validate algorithm and its generalisability clearly requires wider evaluation. On the other hand, the reports were drawn from a long time period (13 years), increasing the potential heterogeneity of raters. The model is also limited in only having been trained on text written in English. Similarly, the overwhelming majority of the reports are addressing the same clinical question. Radiology reports may differ in style dependent on the clinical question and therefore, using the algorithm on both a more generalised or on a different patient population (eg, all outpatient scans, or patients referred from an epilepsy or stroke clinic) would need further evaluation, as the relevant terms of interest may not appear in the report even if present. For example, the radiologist may be less likely to specifically mention the size of the hippocampi for a patient referred from an epilepsy service. This is not necessarily a weakness of the model itself but a limit to its application that needs to be considered in future usage. A further limitation of the model is that it does not seek to capture longitudinal or comparative data. Some of the reports only mentioned that there was no progressive atrophy, implying a consideration of previous reports, and there was no means to evaluate what this meant for the scan in question. Again, this limitation in applicability would need to be considered if the model was used as part of longitudinal research into mild cognitive impairment progression, or evolution of dementia symptoms over time.

## Conclusion

These results support the feasibility and accuracy of natural language processing applied to routine radiology reports and the potential for automated generation of novel meta-data from dementia assessments in electronic health records. This approach is planned to be implemented as part of routine data processing within the CRIS application for future research.

## Supplementary material

10.1136/bmjopen-2024-092459 online supplemental material 1

## Data Availability

No data are available.
