# Supplementary material for: Dementia-related volumetric assessments in neuroradiology reports: a natural language processing-based study
Source: BMJ Open. 2025 Sep 28;15(9):e092459. doi: 10.1136/bmjopen-2024-092459 (PMC12481332; doi:10.1136/bmjopen-2024-092459)
Supplement: online supplemental material 1 [file bmjopen-15-9-s001.docx]

**ANNOTATION INSTRUCTIONS**

There are six categories of interest:

- GVL: ‘Global Volume Loss’
- NO_GVL: ‘No Global Volume Loss
- RVL: ‘Regional Volume Loss’
  NO_RVL: ‘No Regional Volume Loss’
- HVL: ‘Hippocampal / Medial Temporal Lobe Volume Loss’
- NO_HVL: ‘No Hippocampal /Medial Temporal Lobe Volume Loss’

To annotate the text relating to a category (a ‘span’), please click the appropriate category at the top of the window (or use the displayed number shortcut on the keyboard) and then highlight the appropriate words. This is done on a tokenwise basis, so highlighting any portion of the word will include it in the span, so do not worry about being extremely accurate. However, please do not include punctuation at the start/end of a span. e.g. ‘there is generalised volume loss, but no regional atrophy.’, ‘(MTA = 1)’. Spans can overlap, and is often required if two things are mentioned in the same phrase e.g. ‘no regional or hippocampal volume loss ‘ needs the whole span tagged as both NO_RVL and NO_HVL

General rules:

- Do not to make any inferences and base decisions only on what is definite and explicitly documented.
- The span itself should make sense as one of the above categories. If the tagged span reads e.g. ‘left hippocampus’ only then this is likely incorrect
- Do not include leading/trailing parts of a phrase that do not contribute meaning e,g,
  - ‘There is...’ or ‘...is demonstrated’
- Even if there is a clear spelling mistake / VR error, do not tag it
  - e.g. ‘the subarachnoid spaces are globally white’, ‘no lobar trophy’
- Generalised statements that carry no specific information about the above categories are not tagged, e.g.
  - ‘normal intracranial appearances’
  - ‘the brain is structurally normal’
  - ‘no specific imaging pattern’
- Some scans refer to findings in relative terms to a previous study; do not tag anything unless it is clear there is now a definitive positive finding, e.g.
  - ‘there is interval global volume loss’
  - ‘no interval progressive volume loss’ – do not know previous state
  - ‘the degree of sulcal conspicuity appears similar’
  - ‘hippocampal volumes are unchanged’
- Anything similar to ‘appropriate for age’ is a value judgement and should not affect what you tag; tag only what is provided without any judgement e.g.
  - ‘brain volume is normal for age’
  - ‘there is global involutional change appropriate for age’
  - ‘brain volume is appropriate for age’ – unclear, not tagged as anything
- Similarly, ‘in excess of the patients age’ always counts as volume loss, but that part does not need tagging i.e. ‘global parenchymal loss in excess for age’
- Generally, no need to include bilaterality of a finding. Include laterality if abnormal e.g.
  - ‘There is atrophy of the right hippocampal head’
  - ‘left more than right frontal lobe volume loss’
  - ‘the hippocampi are normal in volume bilaterally’
- Include descriptors where they have some indication of severity e.g’ ‘severe hippocampal volume loss’, but do not include vague terms such as ‘some hippocampal volume loss’
- Do not tag mentions of symmetry if otherwise not mentioned as normal/abnormal
  - e.g. ‘the hippocampi are symmetrical’
- Sometimes there are mentions of a feature being ‘without lobar predominance’, please include the mention of the relevant (lack of atrophy) where possible.
  - e.g. ‘generalised volume loss, without lobar or regional predominance’ as RVL
  - (please note ‘generalised volume loss’ would also be tagged as GVL in the above
- Do not tag any part of ‘volume loss in keeping with Alzheimers/frontemporal degeneration/neurodegenerative disorder’ as this refers to a constellation of findings and not a specific volume

Global Volume Loss:

- ‘central white matter volume loss’ is not tagged as any category, as this is a separate process from global atrophy
- Mention of the subarachnoid spaces generally is a euphemism for global volume, e.g.
  - ‘the subarachnoid spaces are maintained’ => NO_GVL
  - ‘there is global prominence of the subarachnoid spaces’ => GVL

Regional Volume Loss:

- Do not tag ‘widening of the sylvian fissures’ as this is not specific enough for regional volume loss (this is sometimes presented as a normal aging or as part of global volume loss)
- If something is very focal and clearly referenced as being a previous injury / stroke using ‘gliosis’, ‘encephalomalacia’ etc then do not tag it. If actually described as ‘volume loss in the inferior frontal gyrus’ then do tag it as RVL.
- Split up references to different areas of volume loss if possible and the spans remain sensible e.g. ‘the is mild widening of sulci and the left temporal pole and prominence of sulci at the left parietal lobe’ can be two separate spans
- Conversely, do not split up regions if they are mentioned en bloc e.g. ‘there is atrophy of the left frontal, right frontal and right parietal lobe’ should only be a single RVL tag
- ‘there is no preferential parietal volume loss’ does not get labelled with anything, as this does not mean that there is no regional volume loss anywhere else

Hippocampal Volume Loss:

- This also includes any other euphemism for hippocampal atrophy e.g.
  - ‘reduction of hippocampal height’
  - ‘flattening of hippocampal head’
- This also includes the medial/mesial temporal lobes
  - ‘anteromedial temporal lobe atrophy’ is HVL
  - NB: ‘widening of the temporal horns’ is also used to describe this
- Tag mentions of MTA score / medial temporal atrophy score / Schelten’s Scale, with ‘MTA 0’ = NO_HVL and everything else (e.g. ‘medial temporal atrophy score of 3’) as HVL
