# Supplementary material for: Dementia-related volumetric assessments in neuroradiology reports: a natural language processing-based study
Source: BMJ Open. 2025 Sep 28;15(9):e092459. doi: 10.1136/bmjopen-2024-092459 (PMC12481332; doi:10.1136/bmjopen-2024-092459)
Supplement: Supplementary file 2 [file bmjopen-2024-092459.reviewer_comments.pdf]

PEER REVIEW HISTORY

BMJ Open publishes all reviews undertaken for accepted manuscripts. Reviewers are asked to complete a checklist review form and are provided with free text boxes to elaborate on their assessment. These free text comments are reproduced below.

ARTICLE DETAILS

Title (Provisional)

Dementia Related Volumetric Assessments In Neuroradiology Reports: A Natural Language Processing Based Study

Authors

Mayers, Adam John; Venkataraman, Ashwin V; Booth, Christopher; Stewart, Robert; Roberts, Angus

---

VERSION 1 - REVIEW

Reviewer **1**

Name **Tummala, Sudhakar**

Affiliation **Department of Electronics and Communication Engineering, School of Engineering and Sciences, SRM University-AP**

Date **14-Nov-2024**

COI **None**

Interesting study, however several points are not clear.

1. The motivation is not very clear. What is the need of NLP here?

2. The NLP pipeline, the model architecture used, how cross-validation was performed not clear. More information on specific pretrained architectures should be given.

3. How are the performance metrics such as recall, precision and F1-score.

4. Since they are medical reports, extracting information should be 100 percent accurate. This study does not achieve that.

5. It would also be more interesting to employ NLP for text summarization of the reports rather than just picking volume variables.

Reviewer **2**

Name **Casey, Arlene**

Affiliation **The University of Edinburgh, Usher institute**

Date **18-Jun-2025**

COI **None**

Thank you for an interesting and well written paper. This feels like a really valuable tool.

I have a couple of minor points and queries that I feel would add some clarification to the paper.

The number of reports you use is 4,700 but in the 'Strengths and /limitations' section you say in brackets more than 3,200 labelled reports. I don't understand what you are trying to say when comparing it to the 4,007. (...of those 4007 3,200 received labels). It is not clear to me so I think you should make this very specific as to why the numbers are different.

Are you able to say something further about the demographics of the records labelled. In particular the age range. I have observed more hedging language used in CT scans for people in lower age ranges. As explained by a clinician this is due to more uncertainty in what is being seen based on the younger age of a patient - I don't know if this applies in this case but it would still be of interest to know if your model's performance is impacted by an age factor and if you think those errors are due to underlying language features, such as uncertainty.

Are the annotation guidelines publicly available (I would encourage you to make this openly available if possible).

Please add a few sentence on the annotation categories that were lower in agreement. I am interested to see how this compared to the model performance inc categories .

Please add a reference for span categorisation for the reader. I feel that some of your audience readers may have never come across this before and may want pointed to a deeper explanation.

On page 10 line 8 you say 'was not considered further in the analysis'. I wasn't a 100% sure if you meant you removed these reports or conflicting labels were not considered further. I think it's the latter but it would be better to be clearer in what you mean.

Can you provide a sentence on whether hedging language caused an issue or discrepancy for the model or annotator agreement.

It is unclear to me what you mean by the last sentence in the conclusion (pg 13 line 19 this approach ...). You are implementing for the NHS as part of there routine processing? You are implementing in your TRE to provide structured data for researchers? It's unclear, please elaborate.

VERSION 1 - AUTHOR RESPONSE

Reviewer: 1
Dr. Sudhakar Tummala, Department of Electronics and Communication Engineering, School of Engineering and Sciences, SRM University-AP

Comments to the Author:
Interesting study, however several points are not clear.
1. The motivation is not very clear. What is the need of NLP here?
NLP is needed where large numbers of documents must be processed in a short timeframe, making manual extraction unfeasible. For example, where there is a requirement to process all patient records from a healthcare provider for audit purposes, or to provide data for cohort based research. Large cohorts from routine healthcare records platforms are already being assembled and analysed for dementia outcomes (e.g. https://doi.org/10.1093/ageing/afac098, https://doi.org/10.1093/ageing/afaf154). Case sample sizes are often in the 10,000's and many will have neuroimaging reports in text format that cannot currently be used for research but would be potentially hugely informative, complementing clinical information.

2. The NLP pipeline, the model architecture used, how cross-validation was performed not clear. More information on specific pretrained architectures should be given.
Additional references for the SpaCy architecture have been included. The transformer model and pipeline components are described in the section titled 'Model Development' with a reference provided for the source of the pre-trained SpaCy pipeline. A Github DOI has been included for the custom k-fold cross validation scripts and the SpaCy pipeline configuration YAML files. Location: Methods

3. How are the performance metrics such as recall, precision and F1-score.
These results are in the abstract and the body of the text. Location: Results.

4. Since they are medical reports, extracting information should be 100 percent accurate. This study does not achieve that.
100% accuracy is unlikely to be feasible with any natural language processing approach, particularly on a dataset which does not have 100% agreement between raters and on a subject matter which does not have 100% agreement between radiologists. Additionally, 100% accuracy is not usually the case for the majority medical tests (an example the reviewer may be familiar with is LFTs). Furthermore, this work is intended to provide data for audit and research, not for direct clinical care, and that accuracy levels can therefore be allowed for in downstream statistical models. A key assumption behind any use of routine healthcare records information is that it is not as accurate as a traditional research study involving recruitment and direct measurement; however, because the samples are much larger and more generalisable than research cohorts, a certain degree of accuracy loss can be accommodated. Clarifications on the objectives and usage of this data has been added. Location: Abstract, Introduction, Conclusion

5. It would also be more interesting to employ NLP for text summarization of the reports rather than just picking volume variables.
The purpose of this model to extract discrete data from the free text reports, for use in research and potentially for onward use in other models. Summarising them would generally not be required as in the clinical setting the clinician seeing the patient would read the

(usually brief) report themselves. Clarifications on the objectives of this study have been added. Location: Abstract, Introduction, Conclusion

Reviewer: 2
Dr. Arlene Casey, The University of Edinburgh
Comments to the Author:
Thank you for an interesting and well written paper. This feels like a really valuable tool.

I have a couple of minor points and queries that I feel would add some clarification to the paper.

The number of reports you use is 4,700 but in the 'Strengths and /limitations' section you say in brackets more than 3,200 labelled reports. I don't understand what you are trying to say when comparing it to the 4,007. (...of those 4007 3,200 received labels). It is not clear to me so I think you should make this very specific as to why the numbers are different.

The full corpus of labelled reports was 4007, with 3200 used for the training of the model and the rest kept as the holdout test set. The body of the text has been updated to clarify this. Location: Methods, Discussion

Are you able to say something further about the demographics of the records labelled. In particular the age range. I have observed more hedging language used in CT scans for people in lower age ranges. As explained by a clinician this is due to more uncertainty in what is being seen based on the younger age of a patient - I don't know if this applies in this case but it would still be of interest to know if your model's performance is impacted by an age factor and if you think those errors are due to underlying language features, such as uncertainty.
Thank you for your very useful comment. However the age of the patient for each report was not included in this dataset and the difference in performance over age ranges is not possible to assess at this time, but this would be of interest to future work

Are the annotation guidelines publicly available (I would encourage you to make this openly available if possible).
These will be added as supplementary data.

Please add a few sentence on the annotation categories that were lower in agreement. I am interested to see how this compared to the model performance inc categories .
The discussion of the relative underperformance of the regional volume loss category has been expanded to make the apparent reasons for this more clear (i.e. that it is a much more heterogenous category than any of the others). Location: Discussion

Please add a reference for span categorisation for the reader. I feel that some of your audience readers may have never come across this before and may want pointed to a deeper explanation.

“Additional references for span categorisation have been included. Location: Methods (technique)”

On page 10 line 8 you say 'was not considered further in the analysis'. I wasn't a 100% sure if you meant you removed these reports or conflicting labels were not considered further. I think it's the latter but it would be better to be clearer in what you mean.
It is the latter of these and the text has been clarified. Location: Methods

Can you provide a sentence on whether hedging language caused an issue or discrepancy for the model or annotator agreement.

Hedging language (e.g. ‘global volume loss appropriate for the patient’s age) was an issue that was decided on at the annotation guideline level, with further clarity provided in the attached annotation guidelines. Essentially, any references to the patient’s age were ignored, so ‘global volume loss in keeping with the patient’s age’ would have ‘global volume loss’ categorised as such. However ‘brain volume is normal for the patient’s age’ would have ‘brain volume is normal’ categorised as such. This has been updated in the body of the text. Location: Methods

It is unclear to me what you mean by the last sentence in the conclusion (pg 13 line 19 this approach ...). You are implementing for the NHS as part of there routine processing? You are implementing in your TRE to provide structured data for researchers? It's unclear, please elaborate.

This has been updated to reflect that this data is for use within CRIS by researchers (and is not in clinical usage). Location: Conclusion

---

VERSION 2 - REVIEW

**Reviewer**	**2**
**Name**	**Casey, Arlene**
**Affiliation**	**The University of Edinburgh, Usher institute**
**Date**	**19-Aug-2025**
**COI**	

Thank you for taking the time to address my comments clearly.
